# Life Style and Sustainable Development

**Published:** 2017-01

**Authors:** Dariush D. FARHUD

**Affiliations:** 1. School of Public Health, Tehran University of Medical Sciences, Tehran, Iran; 2. Dept. of Basic Sciences/Ethics, Iranian Academy of Medical Sciences, Tehran, Iran

## Introduction

Growth and development are the permanent goals of various societies. But, there is a question: How can we access to development? After World War II, the improvements of social and economic situation were followed as a major goal. Therefore, the developed countries find the way of development and innovation in economical development, equality and social justice. According to this viewpoint, development occurs when social and economic development is sustained.

In the last decades, sustainable economic development is more attended in most scientific societies. Social, political and economic sciences as well as biosciences are more focused on sustainable development and its effective factors. Sustainable development is an organized factor that leads to keeping renewable and restricted recourses on earth (1).

The most common definition of sustainable development is: “Sustainable development is development which meets the needs of the present, without compromising the ability of future generations to meet their own needs” (2).

**Fig. 1:**
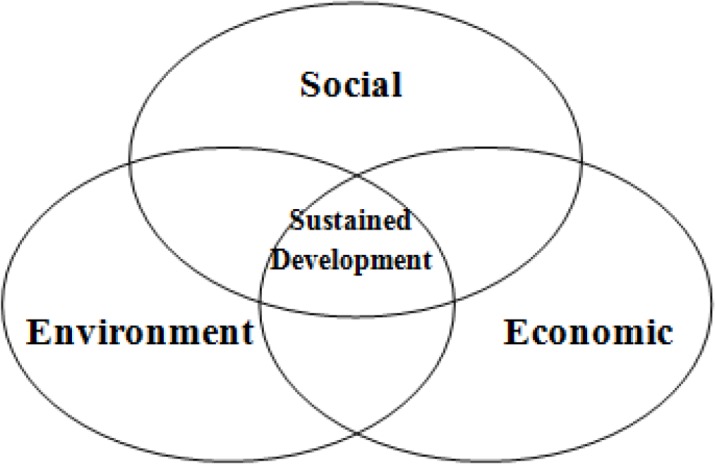
Key predictors of sustained development (5)

According to this definition, various predictors can be imagined for sustainable development: poverty, health, education, demographic characteristics, environmental and natural factors, economical development, climate, national production as well as happiness or well-being (3, 4). Traditionally, economic, social and environmental developments are the most important predictors of sustainable development. These factors have a tight relation with each other (Fig. 1).

1. Economic: sustainable economy leads to increase of productions, better and useful services for the population and it decreases imbalance of economy.
2. Society: sustainable society has the following features: justice, useful social services, gender equality, political stability, security and cooperation.
3. Environment: sustainable environment leads to keeping and maintaining of resources and prevention of wasting renewable resources (6).

A great content affect all three key factors called life style. Life style was attended by researchers and economic experts in 1950s (7). This content provides a life model created by people, groups, governments and societies. Life style depends on citizens and cultural characteristics of a country. Today and with globalization, life style cannot be considered local, but time-orientation is more focused. It is clear that life style can be in the form of political, economical, cultural and religious context (8).

It is necessary to provide a clear definition of life style for understanding sustainable development. There are various definitions; some of them are as followed:

1. Life style is the way of life by people, families and societies.
2. Life style is a set of behaviors that is presented by people, families and societies in different situations (physical, psychological, social and economical).
3. Life style is a branch of habits that includes social fundamentals (9).

Also, various factors are presented for life style. Edward Fern divides all factors in three categories:

1. Actions (jobs, hobbies and funs)
2. Communications (families and friends)
3. Believes (political, religious, social) (10).

Therefore, it can be said that life style includes distinct but clear ways of living that is determined in actions, communications and believes. We believe that life style may leads to sustainable development by impacts on national capitals. Keeping and saving cultural capitals (Antiquities), economic (oil reserves, precious metals, mines and...), social and humanity (family, juveniles, scientists and researchers), ethics (conscientious work, responsibility), environment and physical (industrial machines, materials and buildings), are various forms of national capital and so important in sustainable development. The clear way in keeping and saving the national capitals is the attention to life style.

### Life style and cultural capital

Cultural capital is a concept in sociology and cultural studies. Some of researchers divide cultural capital in three parts:

1. Mental capitals include ideas, theories and great personalities.
2. Visual capitals include cultural products like, books, dictionaries, pictures, drawings…
3. Institutional capitals include academic cultural capital like, finding an academic basis for an idea (11).

Cultural capital is a factor that shapes life style and affected by life style. For example, when national identification of citizens is fading, the governors encourage a kind of life style that people ignore preservation of cultural heritages. In this society, the museums, galleries and all of the antiquities will be destroyed.

### Life style and economic capital

Life style has a significant impact on economic capitals. For instance, consumerism as an inadequate life style is a global problem. By consumerism, each society waste times, money and energy. Although consumerism is an encouraged political way, it is a factor to waste economical capitals (12).

### Life style and human & social capitals

Unhealthy life style threatens human & social capitals. Inadequate life style replaces human values with anti values and then, social relationship networks are interrupted. With unhealthy life style, mental & physical health as the forces of development and advancement, encounter serious challenges (13).

### Life style and ethics capital

Ethics capital is a neglected part of national capital (14). Ethics capital is defined as a complex of ethics values and ethical characteristics of each one in society. Ethics capital has a clear direct and indirect impact on financial aspects (14). The financial aspects of ethics capital are not everything. The improvement of characteristics humanity depends on ethics capital. Ethics capital includes 4 categories:

1. Ethics integrity
2. Ethics Norms
3. Critical ethics
4. Religious ethics (14).

Today, promoting immoral life style and fading of moral values are the significant challenges of societies that should be considered. For example, when family as a first place of forming life style did not train work ethics to children, it leads to irresponsibility in behaviors, deception, wasteful and so on. Society need to learn responsibilities and work ethics to achieve sustainable development. this is possible based on education.

### Life style and environmental capital

Environmental capitals include:

1. Natural environments: jungles, seas, oceans, rivers and so on.
2. All living creatures: animals, birds, plants, trees and so on.
3. Mines and oil and gas reserves (15).

Human beings with improper life styles in environmental capital (contaminated water, soil, and climate, indiscriminate hunting, cutting trees …) destroy biological capitals. Biologists use the concept of Green life style; it shows that life style has significant impact on environmental capital.

### Life style and physical capital

Physical capital points to properties and products of humans. The use of physical is for differentiation between physical and human capitals (16). Examples of physical capitals: public places, buildings like, schools, hospitals, towers, offices and so on.

Damage to each of these instances can destroy physical capital and national capitals. There are some clear examples of inadequate behaviors against physical capitals in our country, Iran:

1. Damaging public properties.
2. Advertising in unauthorized places
3. Writing slogans on the walls

These behaviors damage physical capitals. Lifestyle modification is an important factor for preventing wasting capitals.

## Conclusion

Healthy lifestyle is an important prerequisite for sustainable development. Adequate lifestyle with significant impacts on national capitals like economical, social, cultural capitals can prepare the background for development in social, economical and environmental aspects. Therefore, one of the best ways of keeping and developing national capital is positive attention to lifestyle.

## References

[1] Bossel H (1999). Indicators for sustainable development: theory, method, application. International Institute for Sustainable Development, Canada, pp. 2– 10.

[2] World Commission on Environment and Development (1987). Our common future. Oxford University Press, Oxford, p. 27.

[3] United Nations (2007). Indicators for sustainable development: guideline & methodologies. 3rd Ed United Nations Publication, New York, pp. 10– 18.

[4] Farhud DD Malmir M Khanahmadi M (2015). Increase of individual & social happiness. Iranian Academy of Medical Sciences, Tehran, p. 135.

[5] Gennari P (2007). Key indicators of sustainable development. http://www.mofa.go.jp/policy/economy/eismap/k_seminar/Round-2-3.pdf.

[6] Harris JM (2001). Basic principles of sustainable development. Island Press, Washington DC.

[7] Kirishnan J (2011). Life style: a tool for understanding buyer behavior. Int J Econs Mgmt, 5( 1): 283–298.

[8] Farhud DD Malmir M Khanahmadi M (2015). Indicators of Healthy Lifestyle. Iranian Academy of Medical Sciences, Tehran.

[9] Jensen M (2007). Defining lifestyle. Environ Sci, 4( 2): 63–73.

[10] Fren EF (2001). Advanced focus group research. Sage publication, London.

[11] Bourdieu P (1986). The forms of capital. In: handbook of theory and research for the sociology of education. Eds, Richardson J. Greenwood, New York, pp. 241– 258.

[12] Barin E (2009). Process of consumerism in developing countries. www.khamenei.ir. [In Persian].

[13] Farhud DD (2015). Impact of lifestyle on health. Iran J Public Health, 44( 11): 1442–1444. 26744700 PMC4703222

[14] Bull M Ridley–Duff RJ Foster D Seanor P (2010). Conceptualizing ethical capital in social enterprises. Social Enterprises J, 6( 3): 250–264.

[15] Va Voora A Venema HD (2008). The natural capital approach. International institute for sustainable development. www.iisd.org/pdf/2008/natural_capital_approach.pdf.

[16] Grier R (2005). The interaction of human & Physical capital accumulation: evidence from sub-Saharan Africa. KYKLOS, 58( 2): 195–211.

